# Supplementary material for: Reprogrammed tracrRNAs enable repurposing of RNAs as crRNAs and sequence-specific RNA biosensors
Source: Nat Commun. 2022 Apr 11;13:1937. doi: 10.1038/s41467-022-29604-x (PMC9001733; doi:10.1038/s41467-022-29604-x)
Supplement: Supplementary file 1 — Supplementary Information [file 41467_2022_29604_MOESM1_ESM.pdf]

Reprogrammed tracrRNAs enable repurposing of RNAs as crRNAs and sequence-specific RNA biosensors

Liu *et al.*

Supplementary Information for

"Reprogrammed tracrRNAs enable repurposing of RNAs as crRNAs and sequence-specific RNA biosensors"

Yang Liu[1,2], Filipe Pinto[2†], Xinyi Wan[2†], Zhugen Yang[3,4], Shuguang Peng[5], Mengxi Li[2], Jonathan M. Cooper[6], Zhen Xie[5], Christopher E. French[2,7] and Baojun Wang[1,2,3,7*]

[1]College of Chemical and Biological Engineering & Hangzhou Innovation Center, Zhejiang University, Hangzhou 311200, China

[2]Centre for Synthetic and Systems Biology, School of Biological Sciences, University of Edinburgh, Edinburgh EH9 3FF, UK

[3]Research Centre for Biological Computation, Zhejiang Laboratory, Hangzhou 311100, China

[4]Cranfield Water Science Institute, School of Water, Environment and Energy, Cranfield University, Cranfield, MK43 0AL, UK

[5]Center for Synthetic and System Biology, Department of Automation, Beijing National Research Centre for Information Science and Technology, Tsinghua University, Beijing 100084, China

[6]Division of Biomedical Engineering, James Watt School of Engineering, University of Glasgow, Glasgow, G12 8QQ, UK

[7]Zhejiang University-University of Edinburgh Joint Research Centre for Engineering Biology, Zhejiang University International Campus, Haining 314400, China

[†]These authors contributed equally to this work.

[*]To whom correspondence can be addressed (baojun.wang@zju.edu.cn)

Table of Contents

Supplementary Figures 1−12

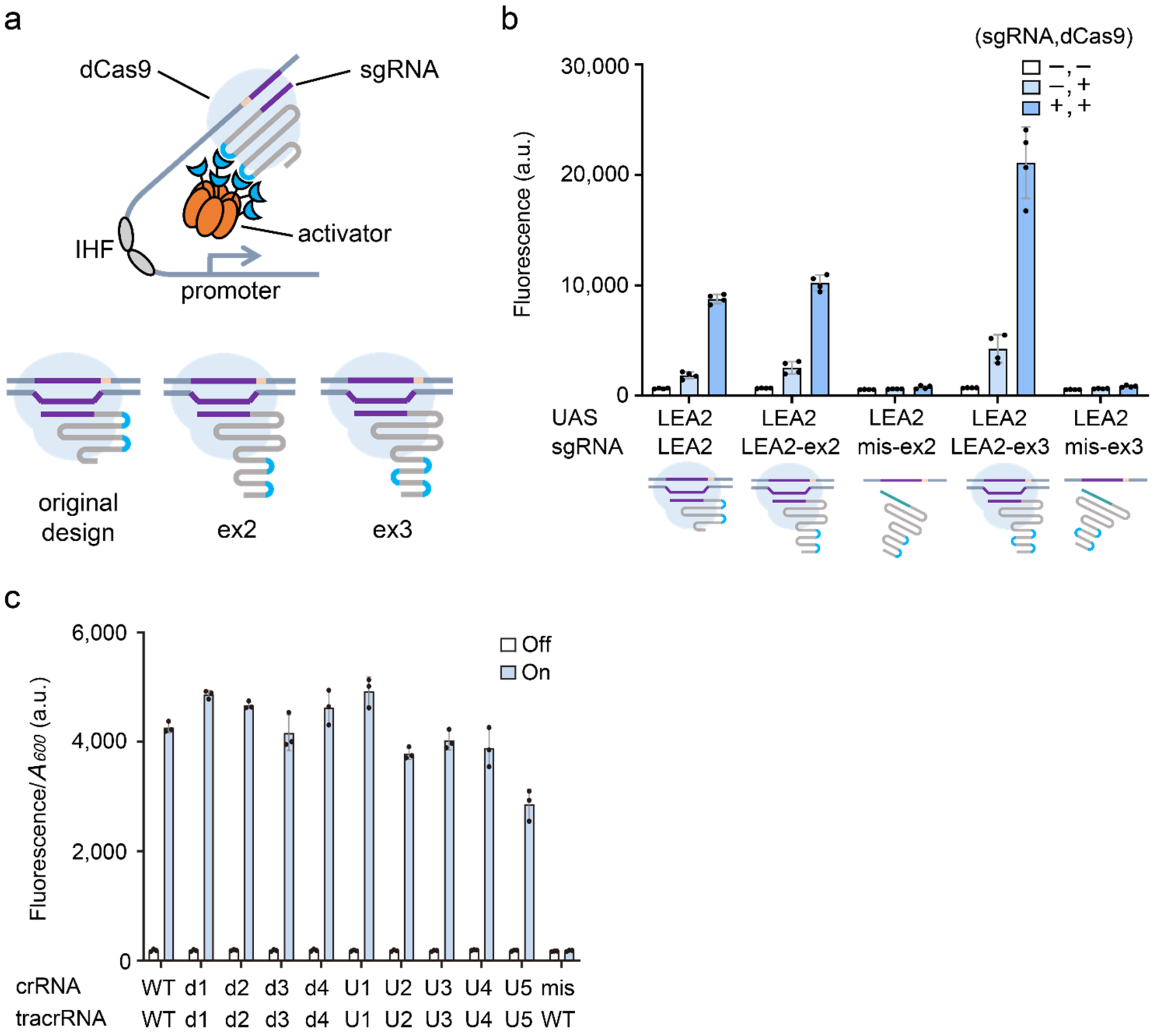

Supplementary Figure 1. CRISPRa function with tail-fused aptamers and CRISPRa output from reprogrammed crRNA-tracrRNA pairs. (a) Schematic of the CRISPRa devices showing three different designs of sgRNA. The light blue U shape line segments in the sgRNA scaffold indicate BoxB RNA aptamers. **(b)** CRISPRa function with three different kinds of sgRNA design. The artificial sequence LEA2 was used as UAS and sgRNA spacer. For each of the two new designs, a sgRNA with the same sgRNA scaffold but with different spacer (artificial sequence LEB3) was employed as mismatched control. dCas9 expression was under control of P_{tet} promoter, and activator expression was driven by a constitutive promoter J23106 (Anderson promoter). A P_{lux2} promoter was used to transcribe sgRNA. 2.5 ng mL^{-1} aTc and 1.6 µM AHL were used for dCas9 and sgRNA induction respectively. Error bars, mean values +/- s.d. (n = 4). **(c)** The CRISPRa function of the various crRNA-tracrRNA pairs in the library shown in **Figure 1e**. The label 'mis' means the WT crRNA has a mismatched spacer (LEB3) with the target UAS (LEA2). All the other crRNAs in this test have spacer LEA2, and a corresponding σ^{54}-dependent promoter with UAS LEA2 was used for the reporter. Expression of dCas9 and activator expression was driven by the P_{tet} and constitutive promoter J23106. A P_{lux2} promoter was used to transcribe sgRNA. No inducer was added for the OFF state. 2.5 ng mL^{-1} aTc, 1.6 µM AHL, and 0.08 mM arabinose were

used for dCas9, crRNA, tracrRNA induction (ON state), respectively. Error bars, mean values +/- s.d. (*n* = 3). a.u., arbitrary units. Source data are provided as a Source Data file.

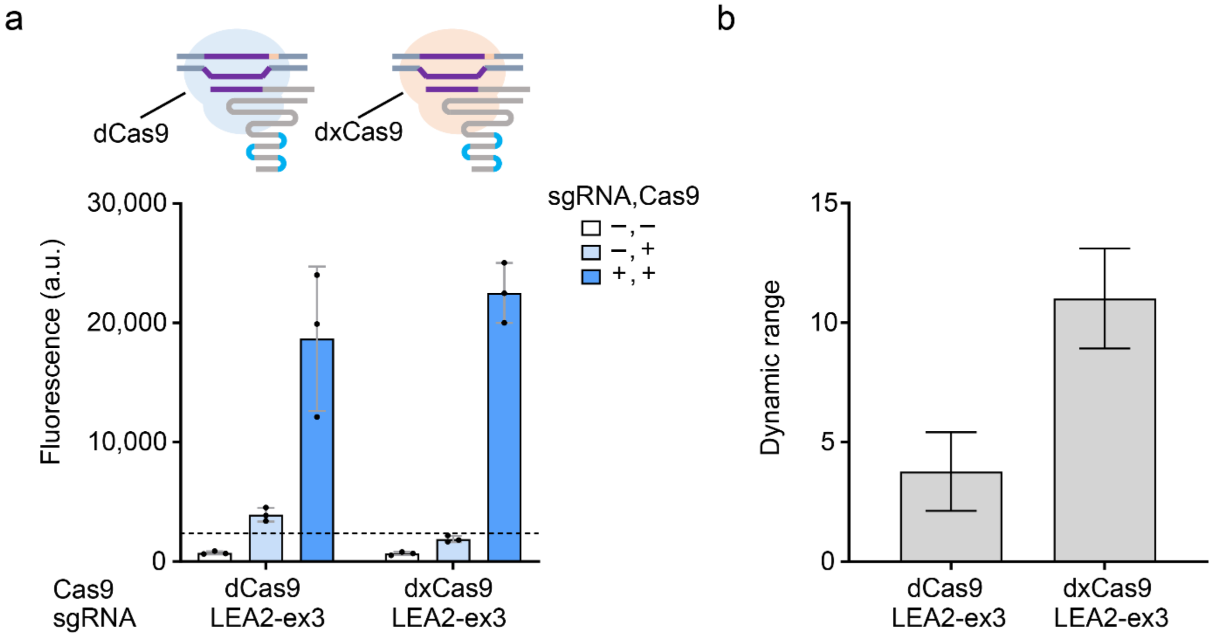

Supplementary Figure 2. sgRNA tuning-based output dynamic range can be improved by employing dxCas9 instead of dCas9. (a) A sgRNA with three BoxB aptamers added to its 3' end tail (LEA2-ex3) was used for CRISPRa. The UAS of promoter and spacer of sgRNA had the LEA2 sequence. P_{tet} controlled dCas9 or dxCas9 generator, and P_{rhaB} drove activator expression, P_{lux2} drove sgRNA transcription. Inducer concentrations: 2.5 ng mL^{-1} aTc, 0.4 mM rhamnose, and 1.6 μM AHL. The plus and minus signs represent the presence or absence of inducers, respectively. Error bars, mean values +/- s.d. (*n* = 3) **(b)** The sgRNA tuning based dynamic range was calculated from the two sets of data with dCas9 or dxCas9 in the left bar chart. Error bars, mean values +/- s.d. (*n* = 3); a.u., arbitrary units. Source data are provided as a Source Data file.

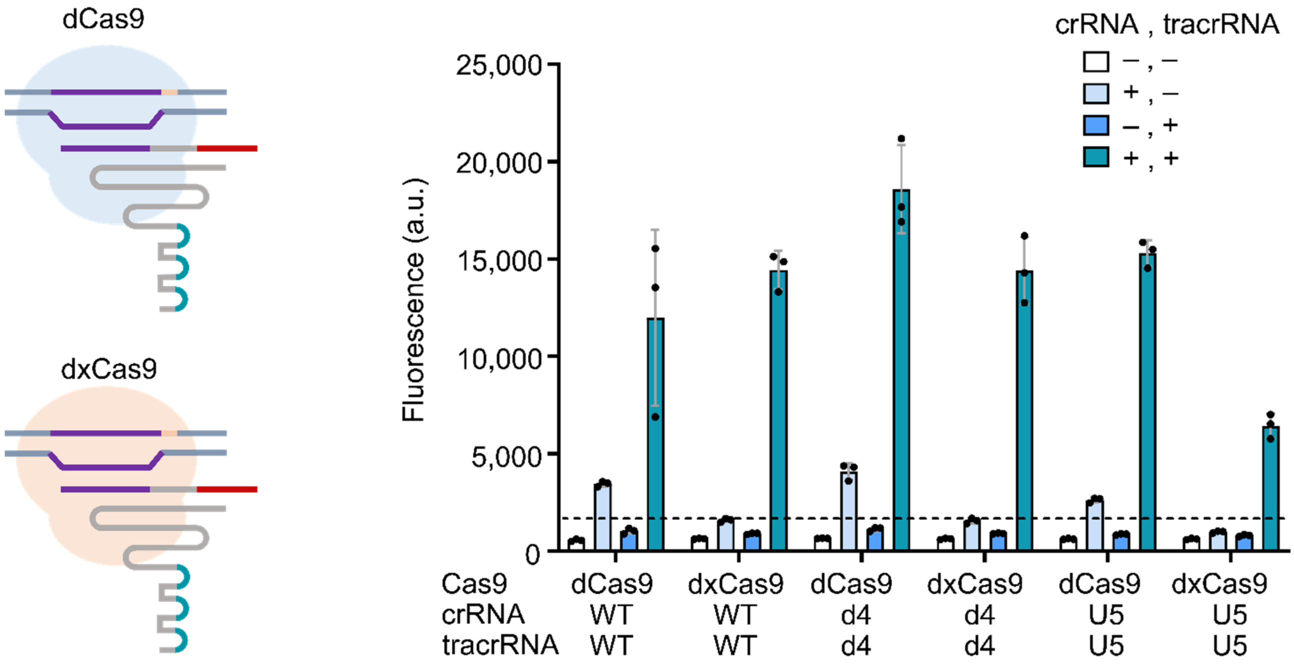

Supplementary Figure 3. Optimizing asymmetry sensitivity to inputs of crRNA-tracrRNA mediated CRISPRa by utilizing dxCas9. The cartoon on the left shows the two CRISPR complexes used in the experiment. The purple line segment represents DNA and its complementary paired spacer sequence of crRNA. The red line indicates the terminator sequence on crRNA. The blue U shaped segment indicates BoxB aptamers at the tail of tracrRNA. The bar chart on the right shows the output of three CRISPRa devices with different crRNA-tracrRNA matching sequences (WT, d4, U5). Artificial sequence LEA2 was used for UAS and spacer in these circuits. Expression of dCas9 or dxCas9 was driven by P_{tet}, and expression of activator was driven by P_{rhaB}. crRNA and tracrRNA transcription was driven by P_{lux2} and $P_{BAD,}$ respectively. Inducer concentrations: 2.5 ng mL^{-1} aTc, 0.4 mM rhamnose, 1.6 μM AHL, and 0.08 mM arabinose. The plus and minus signs represent the presence or absence of inducers. Error bars, mean values +/- s.d. (n = 3); a.u., arbitrary units. Source data are provided as a Source Data file.

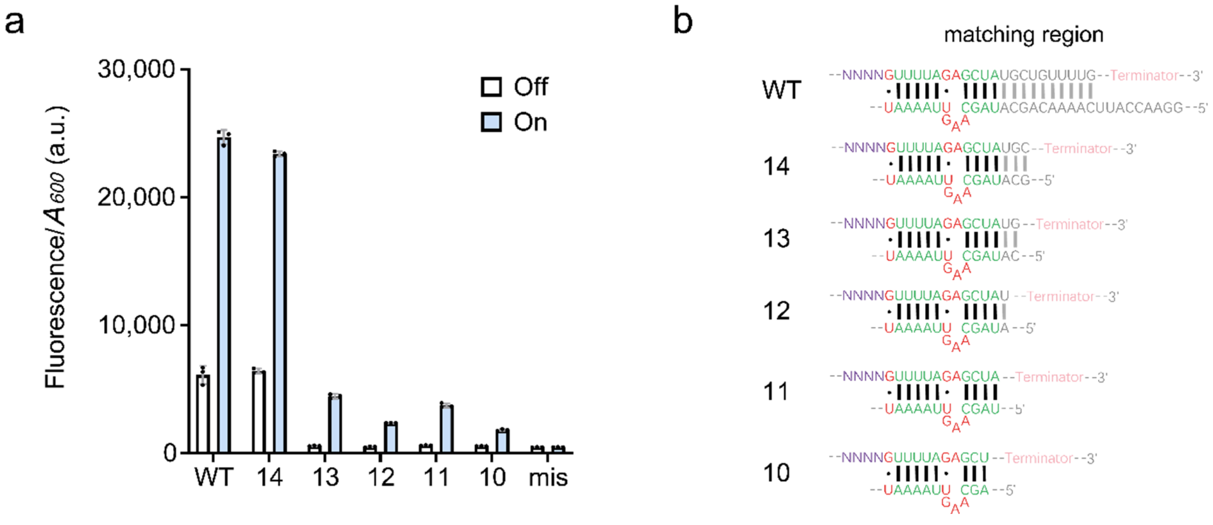

Supplementary Figure 4. Minimal length of the hybridizing sequences required for functional crRNA-tracrRNA pair. (a) CRISPRa output from different lengths of the hybridizing region. The bar chart shows the results from crRNA-tracrRNA pairs with 10 –14 bp length of the matching region. The dCas9 was expressed by P_{tet} promoter with 2.5 ng mL^{-1} aTc. The activator, crRNA and tracrRNA are all driven by the constitutive promoter J23106. The label 'mis' means the WT crRNA has a mismatched spacer (LEB3) with the target UAS (LEA2). All the other crRNAs in this test have spacer LEA2, and a corresponding σ^{54}-dependent promoter with UAS LEA2 was used for the reporter. The data of crRNA-tracrRNA pairs WT, 14 –12 bp, and mis is equal to that in **Figure 2a**. Error bars, mean values +/- s.d. (n = 3); a.u., arbitrary units. **(b)** The structure and sequences of the crRNA-tracrRNA pairs employed in this experiment. Source data are provided as a Source Data file.

a
b
c

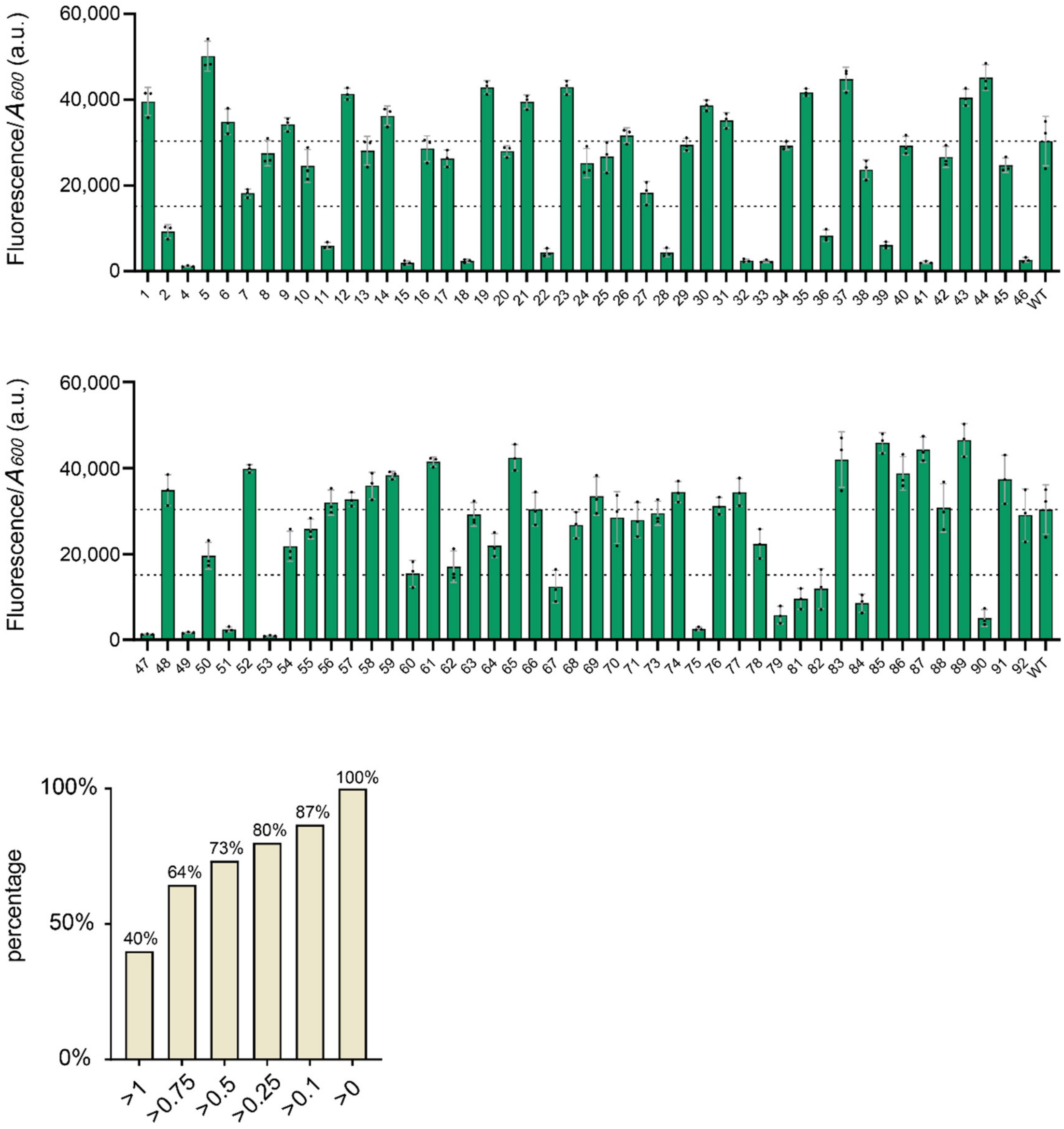

Supplementary Figure 5. CRISPRa output from the paired crRNA-tracrRNA randomized-sequence library. (a) The CRISPRa output from strains 1 – 46 and a positive control strain with the WT crRNA-tracrRNA matching region sequence. The dCas9 generator was controlled by P_{tet}. The crRNA and tracrRNA transcription and activator expression was driven by promoter J23106. 2.5 ng mL^{-1} aTc was used in this test. The dotted lines indicate 100% and 50% output levels from WT strain. Error bars, mean values +/- s.d. (n = 3); a.u., arbitrary units. **(b)** The CRISPRa output from strains 47 – 92 and a positive control strain with the WT crRNA-tracrRNA matching region sequence. The induction condition is the same as in **a**. Error bars, mean values +/- s.d. (*n* = 3); a.u., arbitrary units. **(c)** The percentage of the candidates whose output level is higher than a certain value. The abscissa defines the output range for each group, the values are the different multipliers for output from the WT strain. The corresponding output lower limit is the product of these multipliers and the output value from the WT strain. Source data are provided as a Source Data file.

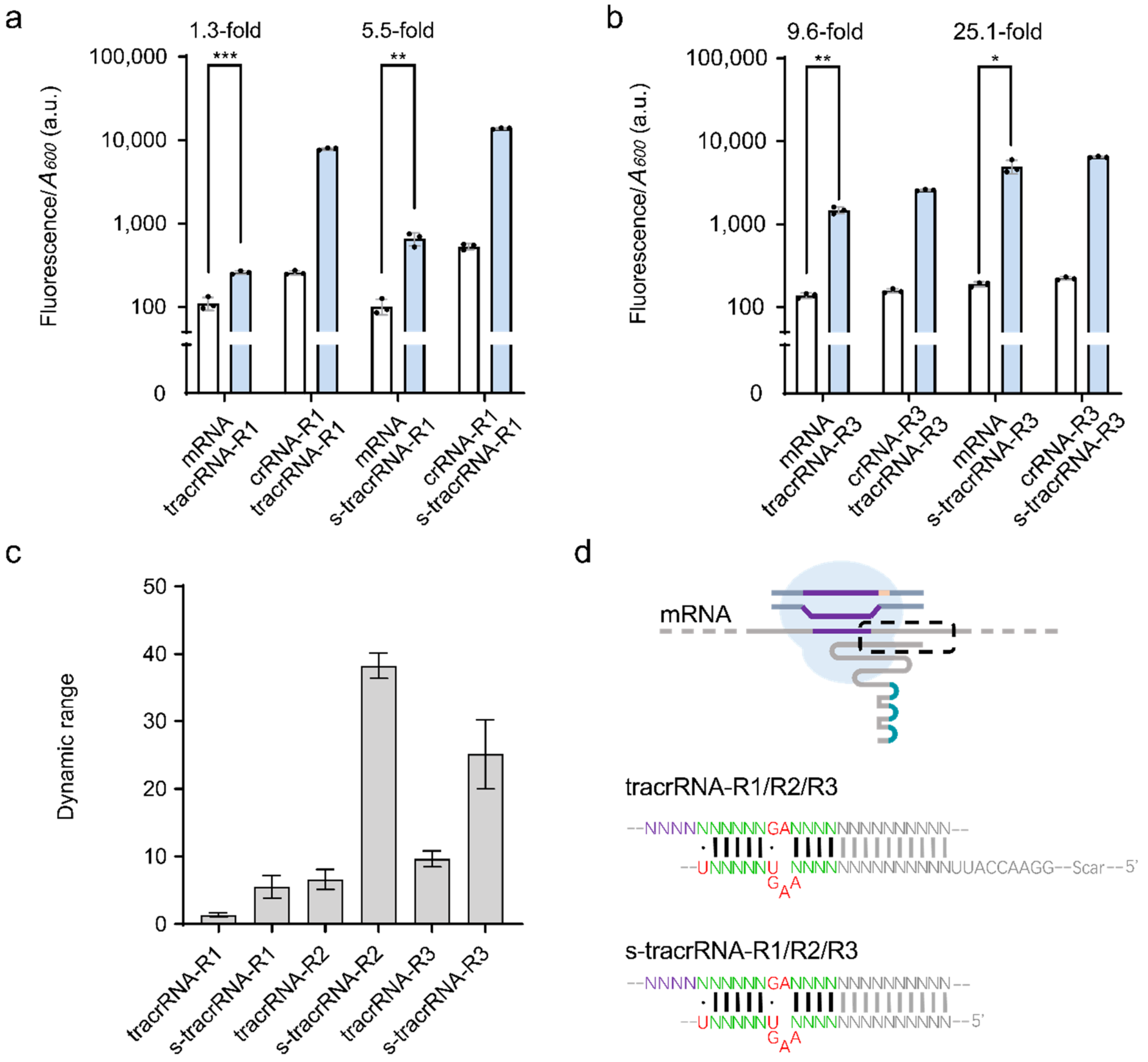

Supplementary Figure 6. Engineered tracrRNA repurposes mRNA into crRNA. (a) 5' end truncation of tracrRNA improves the mRNA-mediated CRISPR function. The mRNA fragment with the same sequences as that in the mRNA target site R1 was expressed as controls. For the bar of mRNA + tracrRNA-R1, the data is the same as in **Figure 3b**. Expression of dCas9 and activator was driven by the P_{tet} and P_{rhaB} promoters, respectively. The mRNA of RFP and the tracrRNA were transcribed from P_{lux2} and $P_{BAD,}$ respectively. Inducer concentrations: 2.5 ng mL^{-1} aTc, 0.2 mM rhamnose, and 0.08 mM arabinose. 0.1 μM AHL was used for mRNA expression. Statistical difference was determined by a two-tailed *t* test: mRNA + s-tracrRNA-R1, $p = 0.0014$, t = 7.904. Error bars, mean values +/- s.d. ($n = 3$). **(b)** The content is the same as in **a**, except that the RNA target is R3. For the bar of mRNA + tracrRNA-R3, the data is the same as in **Figure 3b**. Statistical difference was determined by a Welch's *t* test: mRNA + s-tracrRNA-R3, $p = 0.0121$, t = 8.994. Error bars, mean values +/- s.d. ($n = 3$). **(c)** The dynamic range of different mRNA-mediated CRISPRa devices. All the induction condition is the same as in **a**. Error bars, mean values +/- s.d. (n = 3). **(d)** Schematic diagram shows the structures of the hybridizing regions for the original tracrRNA version and the 5' end truncated tracrRNA. a.u., arbitrary units. Source data are provided as a Source Data file.

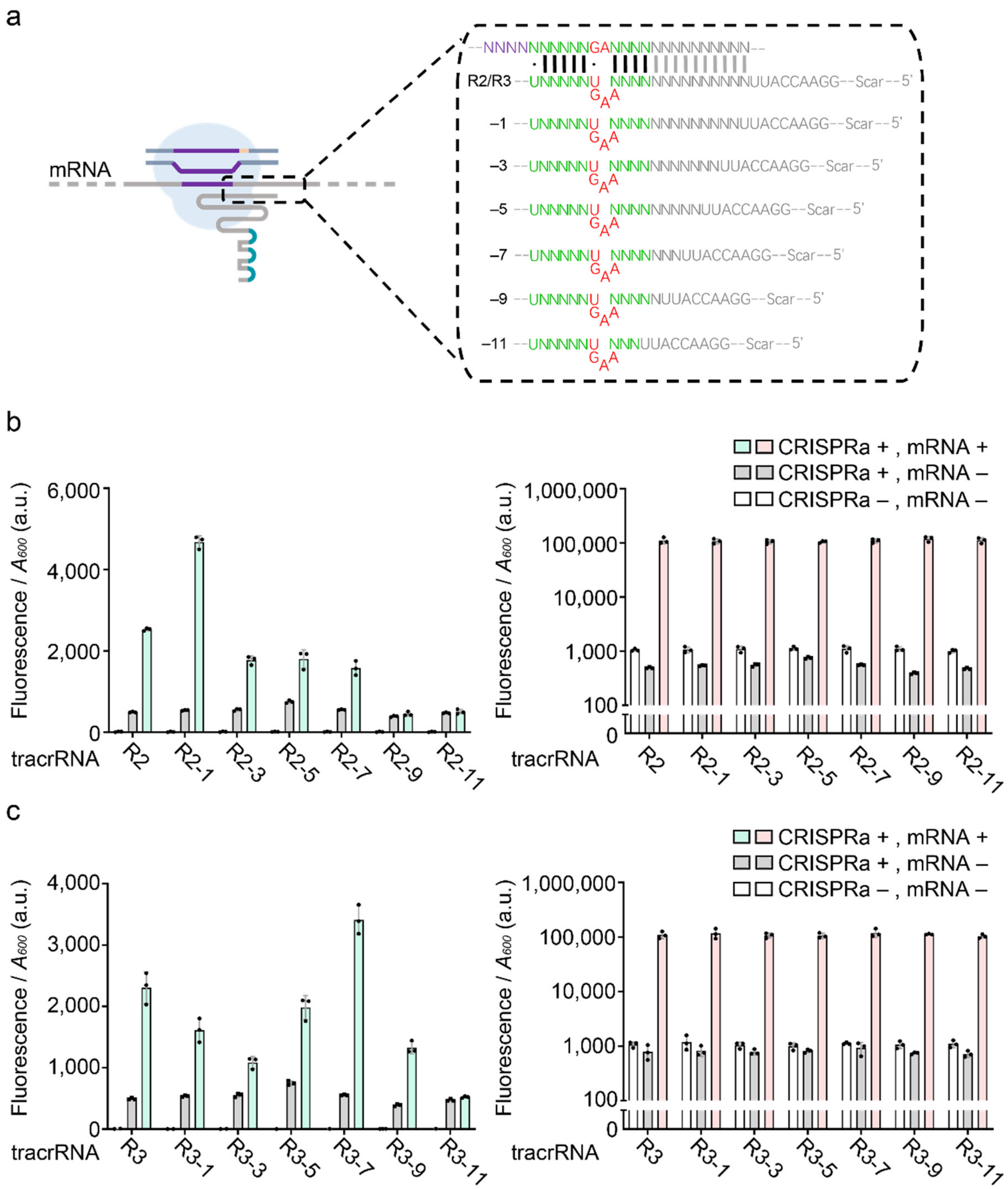

Supplementary Figure 7. Length of the crRNA-tracrRNA hybridizing region affects the CRISPRa function. (a) Schematic showing the design of the library, including truncated tracrRNA anti-coding regions. In this test, tracrRNA-R2 and tracrRNA-R3 were employed and coupled with mRNA of RFP for CRISPRa. The initial pairing length is 21 bp. **(b)** The result of the anti-coding region truncation test on tracrRNA-R2. Green fluorescence detection results on the left and red fluorescence detection results on the right. Expression of dCas9 generator and activator were driven by P_{tet} and P_{rhaB}. mRNA and tracrRNA transcription were driven by P_{lux2} and P_{BAD}, respectively. Inducer concentrations: 2.5 ng mL^{-1} aTc, 1.6 μM AHL, 0.4 mM rhamnose, and 0.08 mM arabinose. CRISPR + or CRISPR – indicates that all the inducers were added or not except AHL (for mRNA induction). Error bars, mean values +/- s.d. (*n* = 3) **(c)** The result of the anti-coding region truncation test on tracrRNA-R3. Green fluorescence detection results are on the left, and red fluorescence detection results are on the right. All the conditions were the same as that in **b.** Error bars, mean values +/- s.d. (*n* = 3); a.u., arbitrary units. Source data are provided as a Source Data file.

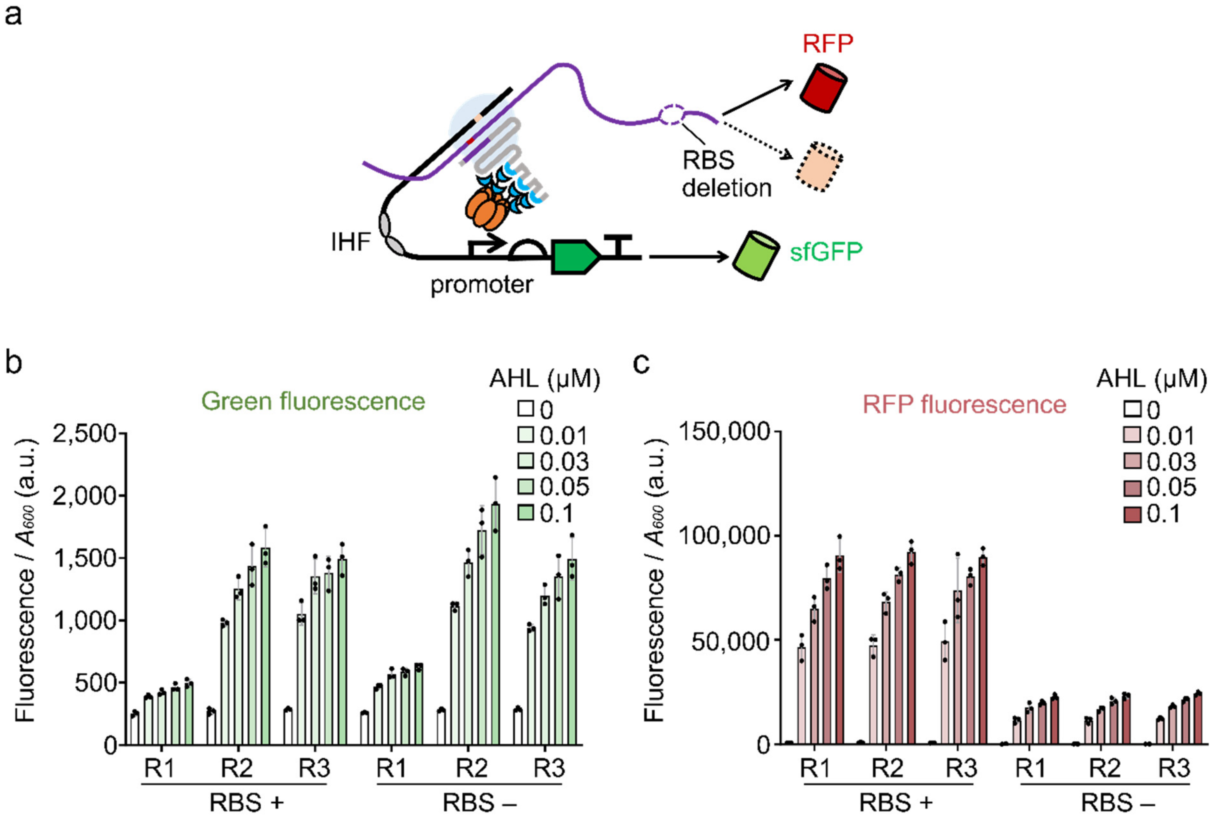

Supplementary Figure 8. Test of ribosome binding site (RBS) deletion in the RFP mRNA hijacking experiment. (a) Schematic showing the circuit design for the RBS deletion experiment. The oval purple dotted line represents the RBS, and the red cylindrical structure drawn by the dotted line indicates that the translation of red fluorescent protein is greatly reduced after the RBS is deleted. **(b)** The green fluorescence output from the mRNA mediated CRISPRa with or without an RBS on the mRNA of RFP. Expression of dCas9 and activator was driven by P_{tet} and P_{rhaB} promoters. mRNA and tracrRNA transcription were driven by P_{lux2} and $P_{BAD,}$ respectively. Inducer concentrations: 2.5 ng mL^{-1} aTc, 0.2 mM rhamnose, and 0.08 mM arabinose. A gradient concentration of AHL (0, 0.01, 0.03, 0.05, 0.1 μM) was used for mRNA induction. Error bars, mean values +/- s.d. (n = 3) **(c)** The red fluorescence output from the mRNA mediated CRISPRa with or without an RBS on the mRNA of RFP. The data was from the same experiment in **b**. Error bars, mean values +/- s.d. (n = 3). a.u., arbitrary units. Source data are provided as a Source Data file.

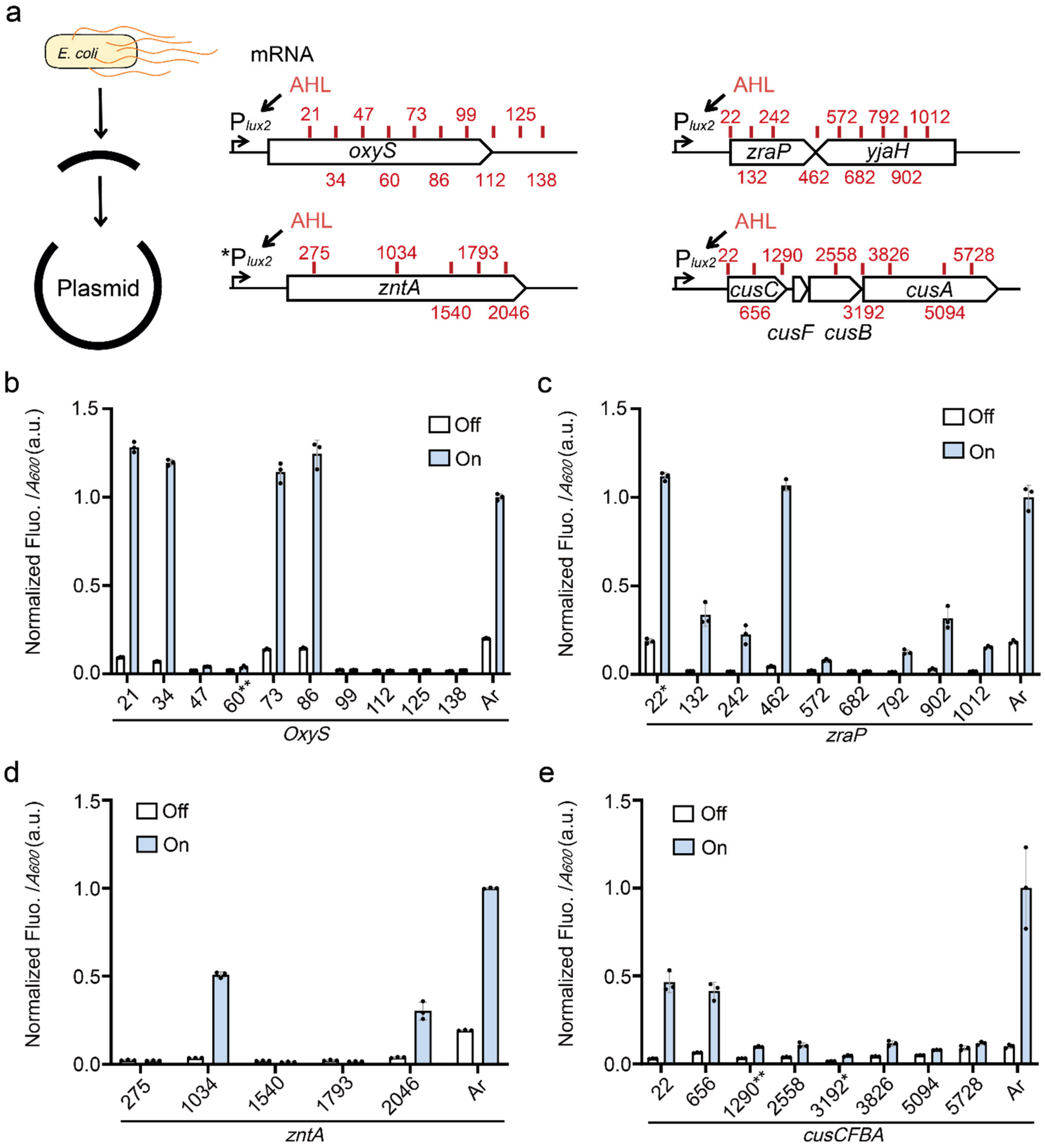

Supplementary Figure 9. Hijacking of small RNA / mRNAs cloned from the *E. coli* genome as crRNAs. (a) Schematic shows the structures of the four environment-responsive genes cloned from the *E. coli* genome. The red line and the corresponding number indicate the tracrRNA binding sites we tested and its distance from the predicted transcription start site. The asterisk indicates that the promoter contains a mutation that was accidentally introduced during construction, which may affect the quantitative relationship of the function of this circuit to the control circuit but does not affect the site screening. **(b)** The CRISPRa output from all tested target sites on the small RNA OxyS and downstream region suspected of being transcribed. The data are normalized with a positive control data from the optimized mRNA hijacking device of the arsenic sensor (described in **Figure 5**). The 'On' indicates that transcription of mRNA was induced using 0.1 μM AHL, and the 'Off' state indicates that no AHL induction was used. The dCas9, activator and tracrRNA were always induced by 2.5 ng mL^{-1} aTc, 0.4 mM rhamnose, and 0.08 mM arabinose, respectively. The double asterisk indicates that the promoter P_{pspA} contains a mutation accidentally introduced during

construction, which is not expected to affect the purpose of the experiment. Error bars, mean values +/- s.d. (*n* = 3). **(c)** The CRISPRa output from all tested target sites on the mRNA of *zraP*. The experimental conditions and data processing method are the same as in **b**. The single asterisk indicates that the reporter gene *sfgfp* contains a mutation (K140N) that was accidentally introduced during construction. Error bars, mean values +/- s.d. (*n* = 3). **(d)** The CRISPRa output from all tested target sites on the mRNA of *zntA*. The experimental conditions and data processing method are the same as in **b**. Error bars, mean values +/- s.d. (*n* = 3). **(e)** The CRISPRa output from all tested target sites on the mRNA of *cusCFAB*. The experimental conditions and data processing method are the same as in **b**. The single asterisk indicates that the reporter gene *sfgfp* contains a mutation (N146S), and the double asterisk indicates that the promoter P_{pspA} contains a mutation accidentally introduced during construction. Both mutations are not expected to affect the purpose of the experiment. Error bars, mean values +/- s.d. (*n* = 3). Source data are provided as a Source Data file.

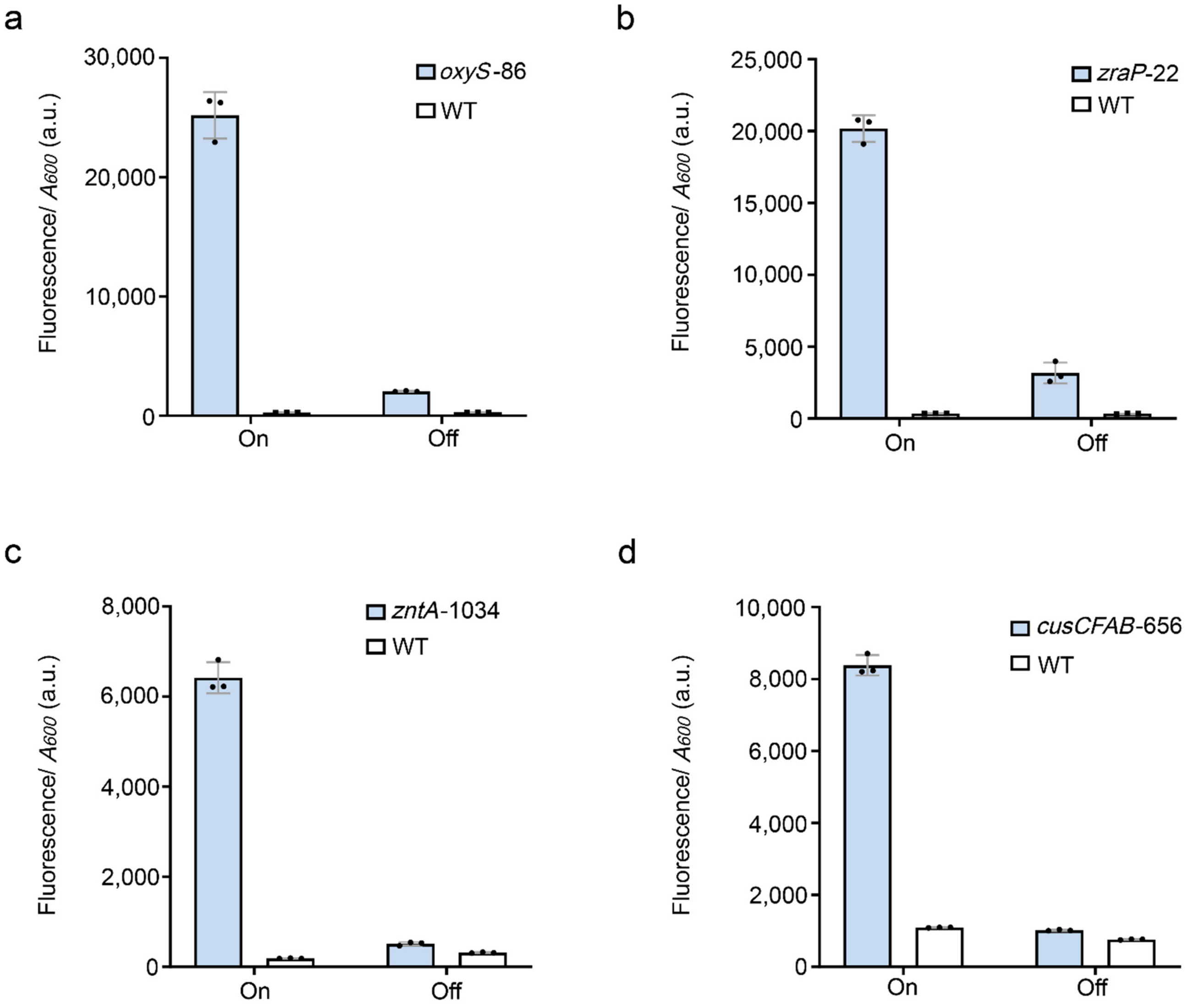

Supplementary Figure 10. Specificity test of the four candidate small RNA / mRNA hijacking devices. (a) The output from the CRISPRa system triggered by small RNA OxyS and the tracrRNA targeting site oxyS-86. A wild type (WT) tracrRNA was employed here as a mis-matched tracrRNA control. The 'On' indicates that transcription of mRNA controlled by promoter P_{lux2} was induced by 0.1 µM AHL, and the 'Off' state indicates that no AHL induction was used. The dCas9, activator and tracrRNA expression were controlled by promoters P_{tet}, P_{rhaB} and P_{BAD}, respectively. And they were always induced by 2.5 ng mL^{-1} aTc, 0.4 mM rhamnose, and 0.08 mM arabinose, respectively. Error bars, mean values +/- s.d. (*n* = 3). **(b)** The output from the CRISPRa system triggered by *zraP* mRNA and the tracrRNA targeting site zraP-22. The experimental conditions are the same as in **a**. Error bars, mean values +/- s.d. (*n* = 3). **(c)** The output from the CRISPRa system triggered by *zntA* mRNA and the tracrRNA targeting site zntA-1034. The experimental conditions are the same as in **a**. The P_{lux2} promoter for *zntA* mRNA contains a mutation that was accidentally introduced during construction, which is not expected to affect the purpose of the experiment. Error bars, mean values +/- s.d. (*n* = 3). **(d)** The output from the CRISPRa system triggered by *cusCFAB* mRNA and the tracrRNA targeting site cusCFAB-656. The experimental conditions are the same as in **a**. Error bars, mean values +/- s.d. (*n* = 3). Source data are provided as a Source Data file.

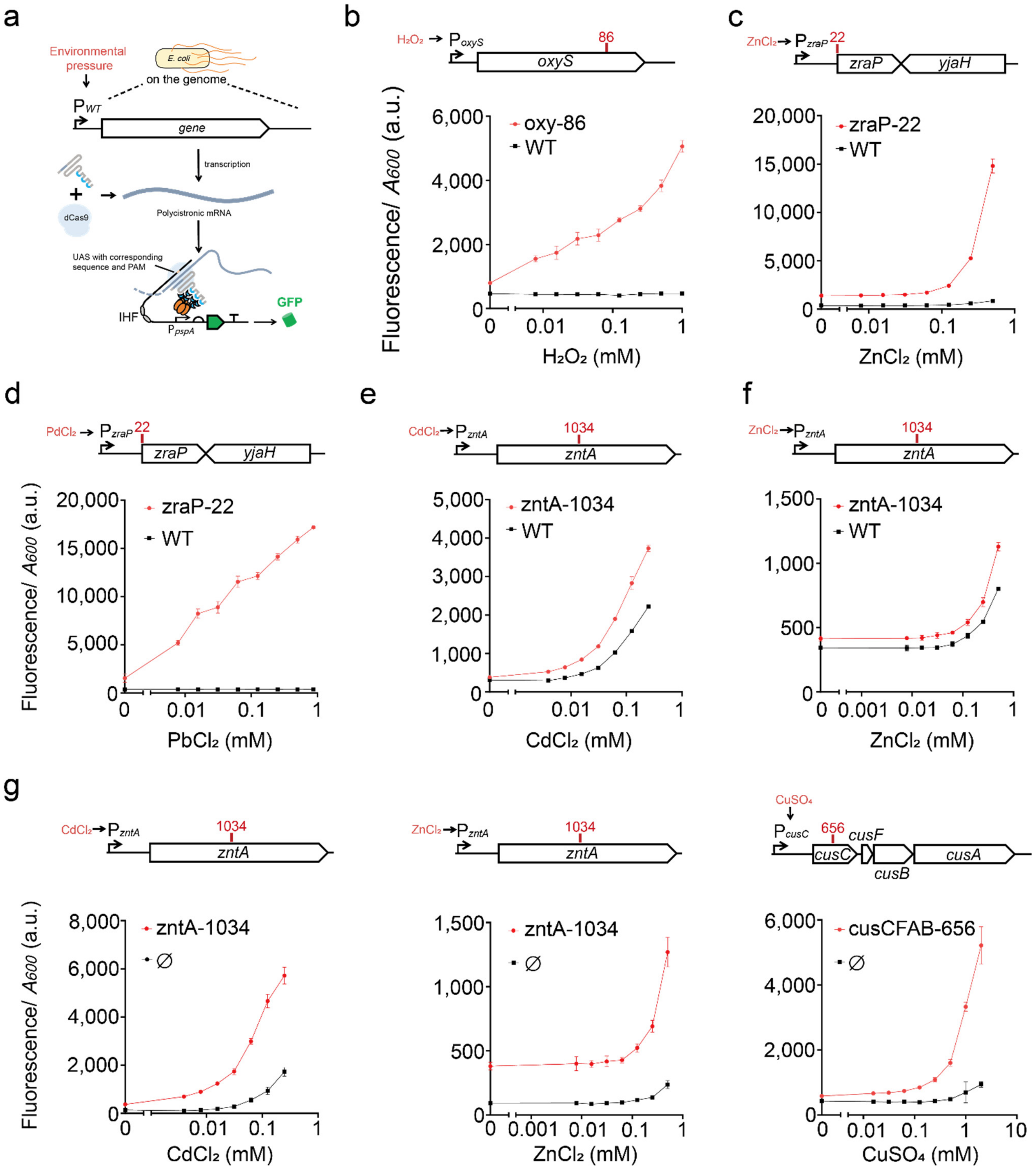

Supplementary Figure 11. Hijacking of endogenous RNAs as crRNAs to detect environmental stress induced transcription. (a) Schematic showing the mechanism of hijacking the endogenous mRNA of the environment-responsive operons of *E. coli* to activate reporter expression by CRISPRa. **(b)** The tracrRNA targeting RNA site oxyS-86 and corresponding promoter were employed to hijack of small RNA OxyS in *E. coli*. A wild type tracrRNA was used as the negative control. Expression of the dCas9 and activator were driven by P_{tet} and P_{rhaB} promoters respectively. The tracrRNA was transcribed from P_{BAD}. Inducer concentrations: 2.5 ng mL^{-1} aTc, 0.4 mM rhamnose, and 0.08 mM arabinose. A gradient concentration of H_2O_2 (0.000, 0.008, 0.016, 0.031, 0.063, 0.125, 0.250, 0.500, 1.000 mM) was used for inducing transcription of the small RNA OxyS. Error bars, mean values +/- s.d. (n = 3). **(c)** The tracrRNA targeting RNA site zraP-22 and corresponding promoter were employed to hijack of mRNA of *zraP* gene in *E. coli*. A wild type tracrRNA was used as the negative control. Expression of the dCas9 tracrRNA, and activator were

driven by the same promoters and induction conditions as in **b**. A gradient of $ZnCl_2$ (0.000, 0.008, 0.016, 0.031, 0.063, 0.125, 0.250, 0.500 mM) was used for inducing transcription of the mRNA of *zraP*. Error bars, mean values +/- s.d. (*n* = 3). **(d)** For the same system described in **c,** a gradient concentration of $PbCl_2$ (0.000, 0.008, 0.016, 0.031, 0.063, 0.125, 0.250, 0.500, 0.870 mM) was used for inducing transcription of the mRNA of *zraP*. Error bars, mean values +/- s.d. (*n* = 3). **(e)** The tracrRNA targeting RNA site zntA-1034 and corresponding promoter were employed to hijack of mRNA of *zntA* gene in *E. coli*. A wild type tracrRNA was used as the negative control. Expression of the dCas9 tracrRNA, and activator were driven by the same promoters and induction conditions in **b**. A gradient concentration of $CdCl_2$ (0.000, 0.004, 0.008, 0.016, 0.031, 0.063, 0.125, 0.250 mM) was used for inducing transcription of the mRNA of *zntA*. Error bars, mean values +/- s.d. (*n* = 3). **(f)** For the same system described in **e**, A gradient concentration of $ZnCl_2$ (0.000, 0.008, 0.016, 0.031, 0.063, 0.125, 0.250, 0.500 mM) was used for inducing transcription of the mRNA of *zntA*. Error bars, mean values +/- s.d. (*n* = 3). **(g)** The empty vector (Ø) was used to replace WT tracrRNA generator as the negative control to reduce the interference by the accumulation of leaky expressed sfGFP. For the data chart on the left, the condition is the same as in **e**, except an empty vector (Ø) was used to replace the tracrRNA generator as the negative control. For the data chart in the middle, the condition is the same as in **f**, except an empty vector as a negative control. For the data chart on the right, the tracrRNA targeting RNA site cusCFAB-656 and corresponding promoter were employed to hijack of mRNA of *cusCFAB* gene cluster in *E. coli*. Expression of the dCas9 tracrRNA, and activator were driven by the same promoters and induction conditions as in **b**. A gradient concentration of $CuSO_4$ (0.000, 0.016, 0.031, 0.063, 0.125, 0.250, 0.500, 1.000, 2.000 mM) was used for inducing transcription of the mRNA of *cusCFAB*. Since the binding of copper ions to biomacromolecules in cells causes changes in basal fluorescence level, the control and data calculation methods for this set of experiments are different from others (see **METHODS**). Error bars, mean values +/- s.d. (*n* = 3). Source data are provided as a Source Data file.

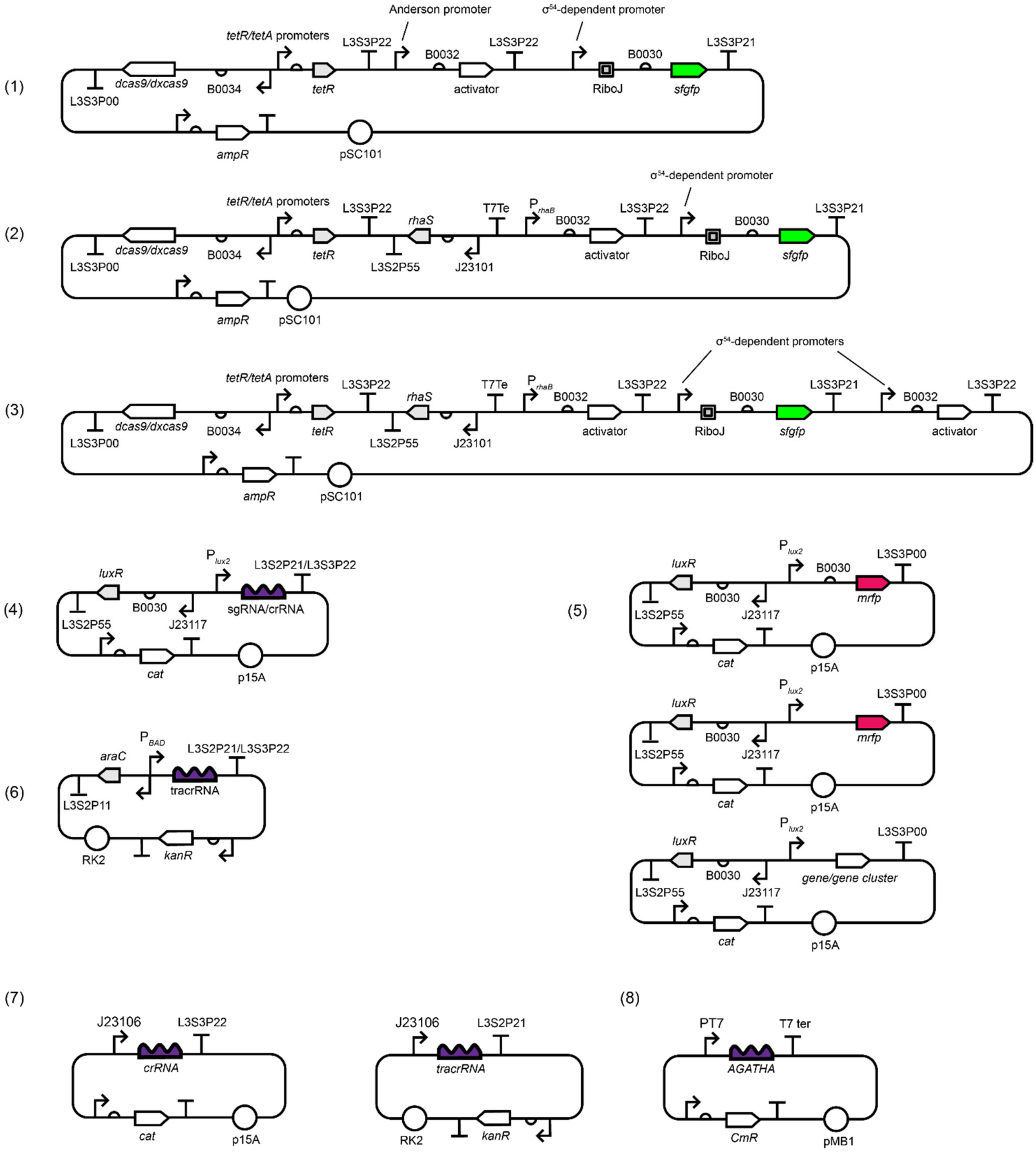

Supplementary Figure 12. Representative plasmid maps for key circuit constructs used in this study. (1) The plasmid map of pLY54. pLY54 was used in the experiments in **Figures 1,2**, and in the tests of **Supplementary Figures 1,4,5**. **(2)** The plasmid map of pLY162−pLY164, and pLY167-pLY169, pLY255, pLY256, pLY295-pLY327. pLY162−pLY164 were used in the RFP mRNA hijacking experiment (**Figure 3**, **Supplementary Figure 6,7,8**); pLY167, pLY168, pLY255, pLY256 are reporter circuits for the hijacking of endogenous mRNA of the arsenic-related gene cluster in *E. coli* (**Figure 4**); pLY169 is the CRISPRa circuit with dxCas9, which was used with pLY327 in experiments of **Supplementary Figure 2,3**. pLY295-pLY326 are reporter circuits used for screening available target sites and hijacking of endogenous small RNA/mRNA in experiments of **Supplementary Figure 9,10,11**. pLY298, pLY305, pLY321, and pLY323 contain mutation that do not affect the purpose of the experiment, which have been marked in **Supplementary Figure 9**

and **Supplementary Data 1**. **(3)** The plasmid map of pLY165 and pLY166, they are positive feedback circuits used in **Figure 3**. **(4)** The plasmid map of pLY76, pLY170−pLY184, pLY187−pLY189, pLY218−pLY231, pLY246 and pLY328-pLY342. They are all the crRNA generators in this study. **(5)** The plasmid map of pLY185, pLY186, pLY221 and pLY259-pLY262. They are mRNA generators with or without RBS in this study. **(6)** The plasmid map of pLY190−pLY217, pLY232−pLY239, pLY251−pLY254, pLY263-pLY294. They are all the tracrRNA generators in this study. **(7)** The plasmid map of pLY241−pLY250, and the 180 crRNA/tracrRNA generators in our library (**Supplementary Data 1**). **(8)** The plasmid map of pLY240, which is an AGATHA circuit **(Figure 5)**.
