# Supplementary material for: Reprogrammed tracrRNAs enable repurposing of RNAs as crRNAs and sequence-specific RNA biosensors
Source: Nat Commun. 2022 Apr 11;13:1937. doi: 10.1038/s41467-022-29604-x (PMC9001733; doi:10.1038/s41467-022-29604-x)
Supplement: Supplementary file 3 — Description of Additional Supplementary Files [file 41467_2022_29604_MOESM3_ESM.pdf]

Title: Supplementary Data 1

Description: Supplementary Tables 1-9 comprising the annotated sequences of the various genetic components, primers and plasmid constructs used in this study, provided as an excel file.

Title: Supplementary Data 2

Description: source data for all plotted figures, provided as an excel file.
