# Supplementary material for: Reprogrammed tracrRNAs enable repurposing of RNAs as crRNAs and sequence-specific RNA biosensors
Source: Nat Commun. 2022 Apr 11;13:1937. doi: 10.1038/s41467-022-29604-x (PMC9001733; doi:10.1038/s41467-022-29604-x)
Supplement: Supplementary file 4 — Reporting Summary [file 41467_2022_29604_MOESM4_ESM.pdf]

nature research

Corresponding author(s): Baojun Wang

Last updated by author(s): Mar 16, 2022

Reporting Summary

Nature Research wishes to improve the reproducibility of the work that we publish. This form provides structure for consistency and transparency in reporting. For further information on Nature Research policies, see Authors & Referees and the Editorial Policy Checklist .

Statistics

For all statistical analyses, confirm that the following items are present in the figure legend, table legend, main text, or Methods section.

n/a	Confirmed	
☐	☒	The exact sample size (*n*) for each experimental group/condition, given as a discrete number and unit of measurement
☐	☒	A statement on whether measurements were taken from distinct samples or whether the same sample was measured repeatedly
☐	☒	The statistical test(s) used AND whether they are one- or two-sided<br>*Only common tests should be described solely by name; describe more complex techniques in the Methods section.*
☐	☒	A description of all covariates tested
☐	☒	A description of any assumptions or corrections, such as tests of normality and adjustment for multiple comparisons
☐	☒	A full description of the statistical parameters including central tendency (e.g. means) or other basic estimates (e.g. regression coefficient) AND variation (e.g. standard deviation) or associated estimates of uncertainty (e.g. confidence intervals)
☐	☒	For null hypothesis testing, the test statistic (e.g. *F*, *t*, *r*) with confidence intervals, effect sizes, degrees of freedom and *P* value noted<br>*Give P values as exact values whenever suitable.*
☒	☐	For Bayesian analysis, information on the choice of priors and Markov chain Monte Carlo settings
☒	☐	For hierarchical and complex designs, identification of the appropriate level for tests and full reporting of outcomes
☐	☒	Estimates of effect sizes (e.g. Cohen's *d*, Pearson's *r*), indicating how they were calculated

Our web collection on statistics for biologists contains articles on many of the points above.

Software and code

Policy information about availability of computer code

Data collection	Omega Control v5.11 R4, Attune NxT Software v3.2.1
Data analysis	Microsoft Excel 2013, Microsoft Excel 2016, Flowjo 7.6.1, FlowJo 10.0.7, Graphpad prism v9.1.1, RNAfold web server (http://rna.tbi.univie.ac.at/cgi-bin/RNAWebSuite/RNAfold.cgi), CLUSTALW 2.1 (https://www.genome.jp/tools-bin/clustalw), mfold Web Server (http://www.unafold.org/), MATLAB 2015b, R 4.0.5, R package 'ggcorrplot'.

For manuscripts utilizing custom algorithms or software that are central to the research but not yet described in published literature, software must be made available to editors/reviewers. We strongly encourage code deposition in a community repository (e.g. GitHub). See the Nature Research guidelines for submitting code & software for further information.

Data

Policy information about availability of data

All manuscripts must include a data availability statement. This statement should provide the following information, where applicable:
- Accession codes, unique identifiers, or web links for publicly available datasets
- A list of figures that have associated raw data
- A description of any restrictions on data availability

All data in the main text and the supplementary materials are provided as a Source Data file. The previously constructed plasmids pLY54(#130923), pLY76(#130963) used in this study are available from Addgene. Representative plasmids of dual-RNA mediated CRISPRa, mRNA hijackers, small RNA hijackers are available from Addgene at https://www.addgene.org/Baojun_Wang/.

Field-specific reporting

Please select the one below that is the best fit for your research. If you are not sure, read the appropriate sections before making your selection.

- [x] Life sciences
- [ ] Behavioural & social sciences
- [ ] Ecological, evolutionary & environmental sciences

For a reference copy of the document with all sections, see nature.com/documents/nr-reporting-summary-flat.pdf

Life sciences study design

All studies must disclose on these points even when the disclosure is negative.

Sample size	Sample sizes in terms of number of replicates are standard in the field, and are described along with Figures. No statistical methods were used to determine the sample sizes. At least 3 biological replicates adhered for all the experiments, which matches the common practices in the field and were such that standard deviations were small enough to allow the determination of significant effects and trends.
Data exclusions	Data that showed aberrant results due to mistakes in experiments executions were excluded. In those cases, experiments were repeated to ensure there were at least three repeats.
Replication	All experiments were performed in three or more replicates. In addition to high-throughput characterization and cell-free experiments, all experiments were biological repeats. For high-throughput characterization and cell-free experiments (Figure 1e, h, i, Figure 2e, f, g and Figure 5), the transformed products or cell-free systems were treated separately for technical repeats. The details are described in the Methods. All attempts at replication were successful.
Randomization	Each biological replicate was inoculated from a single colony, which was randomly chosen from an agar plate. On a single plate, each transformant acquires the same gene circuit. Except for experiments involving high-throughput transformation, the samples used in each set of experiments were independently inoculated with bacteria from a set of plates, and colonies were randomly chosen from each plate. For high-throughput transformation experiments, transformants obtained from the same plasmid sample were distributed equally to parallel liquid culture. In cell-free experiments, the samples to be tested (such as DNA or RNA molecules) are distributed into independent reaction systems.
Blinding	No blinding was involved as it was not necessary in this study. We did not seek to prove or reject any particular claims or hypothesis and so blinding was irrelevant. Additionally, the subject in this study is not humans or animals, which the blinding is not applicable. The experimental conditions were set in parallel, and the bacteria carrying the same circuits generated colonies on agar surface then were randomly selected without subjective factors contributing to bias during this process.

Reporting for specific materials, systems and methods

We require information from authors about some types of materials, experimental systems and methods used in many studies. Here, indicate whether each material, system or method listed is relevant to your study. If you are not sure if a list item applies to your research, read the appropriate section before selecting a response.

Materials & experimental systems

n/a	Involved in the study
[x]	[ ] Antibodies
[x]	[ ] Eukaryotic cell lines
[x]	[ ] Palaeontology
[x]	[ ] Animals and other organisms
[x]	[ ] Human research participants
[x]	[ ] Clinical data

Methods

n/a	Involved in the study
[x]	[ ] ChIP-seq
[ ]	[x] Flow cytometry
[x]	[ ] MRI-based neuroimaging

Flow Cytometry

Plots

Confirm that:

- [x] The axis labels state the marker and fluorochrome used (e.g. CD4-FITC).
- [x] The axis scales are clearly visible. Include numbers along axes only for bottom left plot of group (a 'group' is an analysis of identical markers).
- [x] All plots are contour plots with outliers or pseudocolor plots.
- [x] A numerical value for number of cells or percentage (with statistics) is provided.

Methodology

Sample preparation	Bacterial cells from the 96-well plate culture were transferred and diluted 1 : 100 to another U-bottom 96-well plate with PBS (1 ×, with 1 mg/ml Kanamycin to stop translation). The transferred plate was incubated at 4°C for at least 1 h before the flow cytometry assay.
Instrument	Attune NxT Flow Cytometer and Attune NxT Autosampler.
Software	Attune NxT Software v3.2.1, FlowJo 7.6.1, Graphpad prism 9.1.1, Microsoft Excel 2013, FlowJo 10.0.7
Cell population abundance	At least 10^5 events were collected and recorded, live bacterial cells were then gated by FCS-H and SSC-H based on a fixed interval determined from a control sample of cells with empty vector. Eventually 10^3-10^4 events were gated and analyzed for each sample.
Gating strategy	Live bacterial cells were gated by FCS-H and SSC-H based on a fixed interval determined from a control sample of cells with empty vector. Green fluorescence was detected by BL1 channel (with 488 nm excitation and 530 nm emission). No boundaries between 'positive' and 'negative' cell populations were defined.

☒ Tick this box to confirm that a figure exemplifying the gating strategy is provided in the Supplementary Information.
